# Structures of wild-type and H451N mutant human lymphocyte potassium channel K_V_1.3

**DOI:** 10.1038/s41421-021-00269-y

**Published:** 2021-06-01

**Authors:** Sanling Liu, Yue Zhao, Hao Dong, Liang Xiao, Yong Zhang, Yuqin Yang, Seow Theng Ong, K. George Chandy, Longhua Zhang, Changlin Tian

**Affiliations:** 1 Hefei National Laboratory of Physical Sciences at Microscale, Anhui Laboratory of Advanced Photonic Science and Technology, and School of Life Sciences, University of Science and Technology of China, Hefei, Anhui, China; 2 Kuang Yaming Honors School and Institute for Brain Sciences, Nanjing University, Nanjing, Jiangsu, China; 3 Lee Kong Chian School of Medicine, Nanyang Technological University Singapore, Singapore, Singapore; 4 High Magnetic Field Laboratory, Chinese Academy of Sciences, Hefei, Anhui, China; 5 Shanghai Institute of Materia Medica, Chinese Academy of Sciences, Shanghai, China

**Keywords:** Cryoelectron microscopy, Autoimmunity

Dear Editor,

Voltage-gated potassium channels (K_V_) play vital roles in electrically excitable and non-excitable cells. They usually open with membrane depolarization and allow the flow of K^+^ ions. Ion flow through these channels is curtailed by time-dependent entry into non-conducting inactivated states^1^. Inactivation allows channels to close even in the face of continued stimulation and can occur rapidly (in ms, N-type) or slowly (in s, C-type). This tight governance of ion flow by inactivation is essential for the timing and control functions of K_V_ channels.

K_V_1.3 was the first ion channel discovered in immune cells three decades ago^2^ and exhibited only C-type inactivation^3^. During antigen presentation, the channel clusters at the immunological synapse and promotes Ca^2+^ signaling. Effector memory T cells up-regulate K_V_1.3 during activation^4^. Many toxins from scorpions, sea anemones, and parasitic worms block K_V_1.3 by binding to an external vestibule at the outer entrance to the channel’s pore. Protein engineering of these peptide toxins has resulted in selective K_V_1.3 inhibitors that preferentially suppress proliferation, cytokine secretion, and in vivo migration of effector memory T cells^5^. One inhibitor, dalazatide, advanced to human trials where it ameliorated symptoms in patients with plaque psoriasis^6^. These physiological and therapeutic importance of K_V_1.3 motivated us to elucidate its molecular structure.

To produce a stable homogeneous sample for structural study, we removed residues 1–52 of human K_V_1.3; these residues are absent in mouse and rat K_V_1.3. The channel’s voltage-dependence of activation, use-dependent inactivation, and sensitivity to the K_V_1.3-specific inhibitor ShK-EWSS at low picomolar concentrations (Fig. 1a) matched that of K_V_1.3 in T cells^7^. Since the auxiliary subunit K_V_β2 was reported to promote expression of hK_V_1.3^8^, we expressed hK_V_1.3 with K_V_β2.1, purified the complex, and finally obtained an overall 3.2 Å resolution map (Supplementary Fig. S1). The hK_V_1.3–K_V_β2.1 complex assembled as a tetramer with a four-fold symmetry (Fig. 1b). Each subunit of K_V_1.3 contained a transmembrane domain (TMD) and a cytoplasmic T1 domain, which was a docking platform for the auxiliary β subunit^9^. The TMD consisted of a voltage sensor domain (VSD, helices S0–S4), which responded to changes in membrane potential, and a pore-forming domain (helices S5–S6). The K_V_1.3 T1 domain, helices S5–S6, and K_V_β2.1 were at higher resolution (~2.5–3.5 Å), which allowed for accurate model building. The local resolution for the VSD was between 4 and 5 Å. As a result, most side chains were invisible in these regions. We built the VSD model based on its strong main chain density and the corresponding region in crystal structure of K_V_1.2-2.1 chimera (PDB ID 2R9R)^10^. The final model includes K_V_β2.1 with a NADP^+^ molecule (Supplementary Fig. S2a), which is a cofactor for K_V_β2 subunit^9^, the T1 domain, and the TMD. Human K_V_1.3–K_V_β2.1 complex exhibits overall dimensions of ~140 Å × 100 Å × 100 Å, and the length and width of the TMD are ~55 and ~80 Å, respectively (Fig. 1b). Unsurprisingly, the overall architecture of hK_V_1.3–K_V_β2.1 is remarkably similar to that of rat K_V_1.2-2.1 chimera-K_V_β2^10^ (Supplementary Fig. S2b). By aligning the TMDs of K_V_1.3 and the chimera, we observed a small shift of the K_V_1.3 T1 domain and K_V_β2.1 (Supplementary Fig. S2c), which was also mentioned in the cryo-EM structure of the chimera in nanodiscs comparing to its crystal structure^11^.

**Fig. 1:**
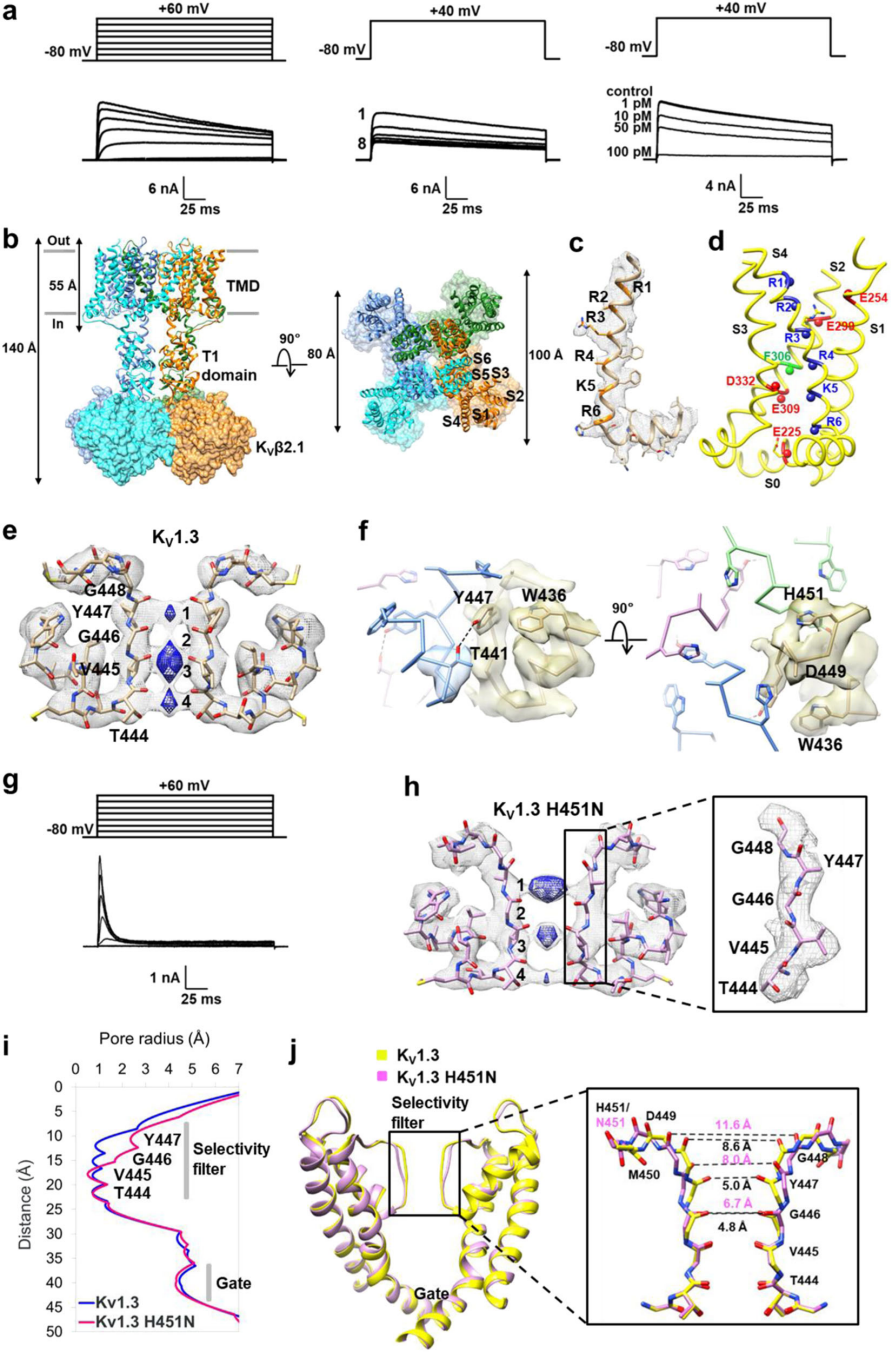
Cryo-EM structure of human K_V_1.3–K_V_β2.1 channel complex. **a** The human K_V_1.3 channel used in the cryo-EM studies (lacking residues 1–52) exhibits properties similar to the native K_V_1.3 channel in human T lymphocytes. Voltage-dependent activation (left), use-dependent inactivation (middle), and block by K_V_1.3-specific inhibitor ShK-EWSS (right). **b** Model of the hK_V_1.3–K_V_β2.1 complex viewed from the membrane plane (left) and from the extracellular side (right). K_V_1.3 subunits are represented as a ribbon, and the K_V_β2.1 subunits as a surface. **c** Density for S4 and the S4–S5 linker in K_V_1.3. Six positively charged residues (R364, R367, R370, R373, K376, and R379) in S4 helix are numbered R1, R2, R3, R4, K5, and R6, respectively. **d** Structure of the K_V_1.3 VSD. The α-carbons of negatively charged and positively charged residues are shown as red and blue spheres, respectively. F306 (green) separates negatively charged residues located at the outer and inner ends of the VSD. The α-carbon of R4 (R373) is at the level of F306. **e** The selectivity filter of K_V_1.3. The blue densities are shown at a higher contour level (0.017) than the gray ones (0.013). **f** The key residues, D449, W436, Y447, and T441, which are expected to be involved in C-type inactivation are shown with the corresponding map. Each subunit is shown in stick with a different color. Potential hydrogen bonding interactions between Y447 and T441 are represented as dashed lines. The distance between oxygen atoms in side chains of Y447 and T441 is 3.4 Å. **g** Voltage-dependent activation of the H451N mutant. **h** The selectivity filter of K_V_1.3 H451N.The blue densities are shown at a higher contour level (0.018) than the gray ones (0.014). **i** Channel pore radius of K_V_1.3 (blue) and K_V_1.3 H451N (pink) calculated using the HOLE program. **j** Structural comparison of the pore-domain in hK_V_1.3 (yellow) and the H451N mutant (pink). Close view of the selectivity filter and distances between carbonyl oxygen atoms of G446, Y447, and G448 from two diagonally opposed subunits are shown for K_V_1.3 and K_V_1.3 H451N, respectively.

In the VSD, the side-chain density for most charged amino acids are not discerned in the map, as was reported for the K_V_1.2 crystal structure^12^. The S4 helix contains six positively charged residues (R364, R367, R370, R373, K376, and R379) that are numbered R1, R2, R3, R4, K5, and R6 in Fig. 1c, d. F306 located near the midpoint of the membrane separates negatively charged residues located at the outer (E254 on S1 and E299 on S2) and inner (E225 on S0, E309 on S2, and E332 on S3) ends of the VSD (Fig. 1d). F306, E309, and D332 form a charge transfer center in the VSD, which facilitates movement of positively charged residues in the S4 in response to changes in membrane voltage^13^. The α-carbon of R4 is at the level of F306, and R1, R2 and R3 are close to the extracellular surface of the channel, consistent with the VSD being in a depolarized conformation. The angle of the S4–S5 linker (Supplementary Fig. S2c) is similar to that of the K_V_1.2-2.1 chimera in open conformation^10^.

In the pore-domain, the selectivity filter and pore helices of K_V_1.3 superimpose well onto that of the K_V_1.2 (Supplementary Fig. S2d). These two structures differ in the loops linking the S5 and S6 helices. These loops form the walls of the external vestibule where toxins bind. These differences in vestibule may contribute to the differing sensitivities of K_V_1.3 and K_V_1.2 to pore-blocking toxins. EM densities corresponding to putative K^+^ ions were observed at ion-binding sites 1, 3, and 4 but not site 2 (Fig. 1e). The cryo-EM map of the K_V_1.2-2.1 chimera embedded in nanodiscs also lacked the K^+^ density at site 2, and one possibility of this absence was suggested to represent a C-type inactivated state^11^. Moreover, side-chain density for Y447 in the filter is weak compared to nearby residues V445 and W436 (Fig. 1f and Supplementary Fig. S2e), and the density corresponding to the side chain of D449 is absent in our map (Fig. 1f). It is possible that the missing side-chain density of D449 is caused by radiation damage. However, it is also possible that an indication of flexibility, which means that Y447 and D449 can adopt various rotameric conformations, suggesting that critical hydrogen bonds related to C-type inactivation between Y447–T441 and D449–W436^14^ may be weakened or broken. These observations suggest that the K_V_1.3 structure may be related to a C-type inactivated conformation.

Earlier studies showed that the mouse K_V_1.3 H404N mutant exhibited very rapid C-type inactivation^15^. We generated the corresponding human K_V_1.3 H451N mutant and tested its electrophysiological properties. The H451N mutant exhibited 60-fold faster inactivation (τ_h_ = 7 ms) than K_V_1.3 (τ_h_ = 440 ms) (Fig. 1g). We determined the cryo-EM structure of the H451N-mutant hK_V_1.3–K_V_β2.1 complex and obtained an overall resolution of 3.3 Å (Supplementary Figs. S3,S4 and Table S1). The overall structures of hK_V_1.3–K_V_β2.1 and hK_V_1.3 H451N–K_V_β2.1 superimpose very well. The main differences between these two structures are in the selectivity filter. The external part of the mutant’s filter is dilated (Fig. 1h, i). In hK_V_1.3’s structure, the carbonyl oxygen atoms of G446, Y447, and G448 in diagonally opposed subunits are 4.8, 5.0, and 8.6 Å apart, which widen to 6.7, 8.0, and 11.6 Å in the mutant, respectively. Under the selectivity filter in both hK_V_1.3 and the mutant, the inner helix bundle forms an open gate, which is sufficiently wide to allow passage of a fully hydrated K^+^ ion.

Close inspection reveals that dilation is not caused only by outward motion of residues in the mutant’s selectivity filter. Moreover, the carbonyl groups of G446 and Y447 rotate and cause their carbonyl oxygen atoms to deviate from the central axis of the pore (Fig. 1j and Supplementary Fig. S5). In K_V_ channels, the precise location of carbonyl oxygen atoms in the filter is essential for selective conduction of K^+^ ions. The deviated carbonyl oxygen atoms contribute to the breakdown of K^+^ coordination at sites 1 and 2. There is no density at site 2, while the density at site 1 is stronger than that at sites 3 and 4 (Fig. 1h). In the dilation model of C-type inactivation, a hydrated Na^+^ has been proposed to replace the K^+^ at site 1 in the C-type inactivated state^1^. The density may be Na^+^, but since K^+^ was the only cation we used during protein purification, the identity of the density at site 1 remains to be clarified.

The stability of the outer pore has been proposed, based on mutagenesis studies of the Shaker channel, to be maintained by a network of hydrogen bonds composed of intra-subunit (W434–D447) and inter-subunit (Y445–T439) interactions^14^. These weaken as the channel opens and rupture as the channel collapses into a non-conducting C-type inactivated state. The corresponding residues in hK_V_1.3 are W436, T441, Y447, and D449. We performed molecular dynamics (MD) simulations to identify the H/N451 site’s effect on the network in hK_V_1.3 and in the mutant. A dominant intra-subunit W436–D449 interaction was observed in hK_V_1.3, together with a secondary H451–D449 inter-subunit interaction. In the mutant, inter-subunit N451–D449 and W436–N451 interactions dominated, with occasional formation of intra-subunit W436–D449 pairs. The hydrogen bonding strengths of the N451–D449 and H451–D449 pairs are comparable according to our density-functional theory calculations, but steric hindrance encountered in the presence of the imidazole ring of H451 limits its rotation compared to the more flexible N451, resulting in the N451–D449 hydrogen bond being favored over H451–D449 (Supplementary Figs. S6 and S7).

Based on the cryo-EM structures and the MD simulation results, we propose that H451N-mutant hK_V_1.3 inactivates rapidly because the pore-stabilizing hydrogen bond network is weakened, leading to external pore dilation. In hK_V_1.3, H451 makes “illicit” interactions with D449 and W436 in neighboring subunits, and these interfere with the hydrogen bond network, rendering the channel more prone to enter the C-type inactivated state. In the rapidly inactivating mutant, N451 makes inter-subunit interactions with D449 and W436 more frequently because it is more flexible than wild-type H451. Consequently, Y447, which located in position close to both D449 and W436 in the same subunit, is possibly destabilized, moves outwards, and rotates away from the pore axis, leading to dilation of the external pore. This deformation limits the ability of carbonyl oxygen atoms of G446 and Y447 to coordinate K^+^ ions at sites 1 and 2.

In summary, we report the cryo-EM structures of human lymphocyte K_V_1.3 channel and a rapidly inactivating mutant H451N. In these two structures, the VSD is in a depolarized conformation and the inner gate is open. Due to the incomplete ion occupancy in the selectivity filter and the weak side-chain density for Y447 and D449, both the wild-type and the H451N-mutant K_V_1.3 structures may be related to C-type inactivated conformation. These two structures provide the basis for guiding the design of new K_V_1.3 inhibitors, which could stabilize the inactivated conformation, for use as immunomodulators.

## Supplementary information

Supplementary information, Figs and Table

## Data Availability

Cryo-EM density maps and atomic coordinates of hK_V_1.3–K_V_β2.1 and hK_V_1.3 H451N–K_V_β2.1 have been deposited in the Electron Microscopy Data Bank under accession number EMD-31148 and 31149, and in the Protein Data Bank under accession code 7EJ1 and 7EJ2, respectively.
