# Supplementary material for: Structures of wild-type and H451N mutant human lymphocyte potassium channel KV1.3
Source: Cell Discov. 2021 Jun 1;7:39. doi: 10.1038/s41421-021-00269-y (PMC8167131; doi:10.1038/s41421-021-00269-y)
Supplement: Supplementary file 1 — Supplementary information, Figs and Table [file 41421_2021_269_MOESM1_ESM.pdf]

Supplementary Information

Materials and Methods

Cloning and protein expression

To improve biochemical stability, residues 1-52 from the N-terminus of human $K_V1.3$ (UniProtKB accession P22001) were removed. This deletion makes the human channel similar to mouse and rat $K_V1.3$, which lack the first 52 residues. DNA encoding $hK_V1.3$ and human $K_V\beta2.1$ (UniProtKB accession Q13303) were cloned into a pFastBac Dual vector (Invitrogen) with a FLAG tag (DYKDDDDK) at the amino terminus of $hK_V1.3$. We used side-directed mutagenesis to introduce the H451N mutation into $hK_V1.3$. All plasmids were sequenced. Recombinant baculoviruse expressing $hK_V1.3$-$K_V\beta2.1$ and $hK_V1.3$ H451N-$K_V\beta2.1$ were generated using the Bac-to-Bac system (Invitrogen). For large-scale expression, baculovirus after two rounds of amplification was used to infect Sf9 cells at 2 x 10^6 cells/ml cultured in SIM SF medium (Sino Biological Inc.) at 27 ℃. Infected Sf9 cells were cultured for 60 h before harvesting.

Protein purification

Cell pellet from 1 L of culture was resuspended in lysis buffer (20 mM Tris pH 8.0, 150 mM KCl, 10% glycerol). The suspension was supplemented with 1.5% (w/v) n-dodecyl-β-D-maltopyranoside (DDM, Anatrace), 0.3% (w/v) cholesteryl hemisuccinate (CHS, Sigma) and protease inhibitor cocktail. After extraction at 4 ℃ for 2 hours, the insoluble fraction was removed by ultra-centrifugation at 180,000 g for 45 min at 4 ℃ and the supernatant was incubated with anti-Flag M2 affinity gel (Sigma) at 4 ℃. The resin was then collected and washed with wash (W) buffer (20 mM Tris pH8.0, 150 mM KCl, 0.1% DDM, 0.02% CHS). The proteins were eluted with W buffer supplemented with 200 μg/ml FLAG peptide. After elution, the proteins were concentrated and further purified on a Superose 6 increase column (GE Healthcare) equilibrated with 20 mM Tris pH 8.0, 150 mM KCl, 0.025% DDM, 0.005% CHS, and 3 mM DTT. Peak fractions of the protein complex were concentrated to ~6 mg/ml and used in cryo-EM experiments.

Cryo-EM sample preparation and data acquisition

3 μl of protein sample at ~6 mg/ml was applied to freshly plasma-cleaned (H_2/O_2, 10s) holey carbon grids (Quantifoil, R1.2/1.3, 300 mesh, Au). The grids were blotted for 5 s at 100% humidity and 4 ℃ with a Vitrobot Mark IV (ThermoFisher Scientific) and plunge-frozen into liquid ethane cooled by liquid nitrogen. The blotted grids were stored in liquid nitrogen until imaged.

For data collection, grids were transferred to a Titan Krios electron microscope (FEI) operated at 300 kV equipped with a Gatan K2 Summit direct detection camera. Movies were recorded in

counting mode with SA29,000x magnification yielding a pixel size of 1.014 Å using the automated image acquisition software SerialEM[1]. Image stacks were obtained with a dose rate of 8 e^-/pixel/s and total exposure time of 8 s with 0.2 s per frame, resulting in a total dose of 62 electrons per $Å^2$. Nominal defocus values ranged from -1.5 to -2.5 μm. Datasets of the $hK_V1.3$-$K_V\beta2.1$ and $hK_V1.3$ H451N-$K_V\beta2.1$ included 1,983 and 2,260 movies, respectively.

Cryo-EM data processing

Dose-fractionated image stacks were subjected to beam-induced motion correction and dose-weighting using UCSF MotionCor2[2]. Contrast transfer function parameters were estimated with Gctf[3]. For particle picking, around 2,000 particles were picked manually to generate templates for auto-picking. The auto-picked particles from Gautomatch (written by Kai Zhang, MRC-LMB Cambridge, UK) were extracted and subjected to reference-free 2D classification in Relion-3.0[4]. For the dataset of $hK_V1.3$-$K_V\beta2.1$, 102,321 particles from well-defined 2D averages were selected and combined for 3D classification. A 3D initial model *de novo* from the 2D average particles was generated using stochastic gradient descent (SGD) algorithm in Relion-3.0. The 50 Å low-pass filtered initial model was used as a reference for 3D classification into three classes. A selected class with continuous density for all transmembrane helices (total 44,078 particles) was used to perform further 3D refinement. 3D refinements with C1 symmetry and C4 symmetry were performed in turn. By comparison with the map resulting from C4 symmetry imposed, the map with C1 symmetry did not display discernible deviations from the C4 symmetry axis. After further particle polishing and CTF refinement, the resulting 3D reconstruction yielded an EM map with a global resolution of 3.2 Å. Dataset of the $hK_V1.3$ H451N-$K_V\beta2.1$ was similarly processed in Relion-3.0, with subset of 79,338 particles producing final maps with global resolution of 3.3 Å. The resolutions were estimated by applying a soft mask excluding detergent micelle and the gold-standard Fourier shell correlation (FSC) using the 0.143 criterion. Local resolution was determined using ResMap[5] with unfiltered half-reconstructions as input maps.

Model building, refinement and validation

Models were built in the software Coot[6] using the crystal structure of rat $K_V1.2$-2.1 chimera-$K_V\beta2$ complex as a reference (PDB ID 2R9R)[7]. $K_V\beta2$, the T1 domain of $K_V1.2$, and the transmembrane domain of the $K_V1.2$-2.1 chimera were fitted into the EM density map of $hK_V1.3$-$K_V\beta2.1$ using UCSF Chimera[8], respectively. The sequences were mutated with corresponding residues in $hK_V1.3$-$K_V\beta2.1$ in Coot. Every residue was manually examined. The initial model was subjected to iterative manual rebuilding in Coot and real-space refinement in PHENIX[9]. The final refinement statistics were validated using the module "comprehensive validation (cryo-EM)" in PHENIX[10]. The N-terminal residues before Gly103, S1-S2 loop (residues 262-292), S3-S4 loop (residues 348-358) and C-terminal residues after Gly503 were not built due to the lack of corresponding densities. The T1-S0 linker (residues 205-211) was built as a poly-alanine chain

based on the weak electron density corresponding to this segment in $hK_V1.3$. The $hK_V1.3$-$K_V\beta2.1$ model was subsequently docked into the EM density maps of $hK_V1.3$ H451N-$K_V\beta2.1$. The model was further subjected to manual rebuilding, real-space refinement and validation. All the figures were prepared using UCSF Chimera.

Electrophysiology studies

DNA encoding $hK_V1.3$ was cloned into a pcDNA3.1/Zeo(+) vector. Site-directed mutagenesis was used to introduce H451N mutation in $hK_V1.3$. All plasmids were sequenced before further study. Expression vectors bearing $hK_V1.3$ (wild-type or mutant) and EGFP were co-transfected into Chinese hamster ovary (CHO) cells cultured in DMEM/F12 (GIBCO) media supplemented with 10% fetal bovine serum (FBS, GIBCO) and 1% Penicillin-Streptomycin-Glutamine (GIBCO) at 37 ℃ in a 5% CO_2 incubator. For each transfection of a 24-well plate well, 1 μg of plasmid encoding wild-type or mutant $hK_V1.3$ and 0.8 μg of plasmid encoding EGFP were diluted into 25 μl of Opti-MEM media (GIBCO). 1 μl of lipofectamine 3000 (invitrogen) was also diluted into 25 μl of Opti-Mem media. These two diluted solutions were mixed for around 30 min and added to the cells. After incubation for 5 h, the cells were transferred to poly-L-lysine-coated slides for culture for another 24-48 h in fresh medium. They were then used for electrophysiological recording.

For whole-cell patch clamp recordings, the bath solution contained 150 mM NaCl, 4 mM KCl, 2 mM $CaCl_2$, 1 mM $MgCl_2$, and 10 mM HEPES (pH 7.4, ~308 mOsm). The electrodes were pulled from thick-walled borosilicate glass capillaries with filaments (1.5 mm diameter, Sutter Instruments) on a four-stage puller (P-1000, Sutter Instruments) and had resistances of 3-5 MΩ when filled with intracellular solution containing 140 mM KCl, 10 mM NaCl, 5 mM EGTA, and 10 mM HEPES (pH 7.4, ~297 mOsm). All chemicals were obtained from Sigma. Experiments were performed at room temperature with an EPC-10 amplifier (HEKA Electronic) using the data acquisition software PatchMaster. Families of $K_V1.3$ and the H451N mutant currents were elicited by voltage steps from -80 mV to +60 mV in 20 mV increments for 200 ms, from the holding potential of -80 mV. To assess use-dependent inactivation, we applied eight voltage steps to +40 mV from the holding potential (-80 mV) once every 2s and measured currents. For pharmacological studies, $hK_V1.3$ currents were elicited by 200 ms depolarization steps applied every 30 s from the holding potential (-80 mV) to +40 mV. After three stable records of currents, ShK-EWSS was applied to the patched cell through a Y-tube perfusion system and the degree of block determined.

Molecular dynamics (MD) simulation

We used our cryo-EM structures of $hK_V1.3$ and $hK_V1.3$ H451N as starting templates for MD simulation. Since the cytosolic T1 domain in the N-terminus should have no direct effect on C-type inactivation[11], we removed it from the structure for our MD simulation studies. Missing loops

in the structure were added using loopy[12]. The resulting channel was embedded in a fully hydrated lipid bilayer of 1-palmytoil-2-oleoyl-snglycero-3-phosphatidylcholine (POPC). The initial position of the protein relative to the lipid bilayers was determined by maximizing the distance between the lipid-facing charged residues and the central plane of the membrane[13]. Molecules within 1.5 Å of protein were deleted to avoid clash. The prepared structure model contains a total number of ~233,700 atoms, including 53,319 TIP3P waters, 353 POPC molecules, 170 K^+ and 146 Cl^- ions. The extra 24 cations served to neutralize the system. Regarding K^+ ions in the selectivity filter, two configurations were tested. In case 1, two K^+ ions occupied the S2 and S4 sites, with S1 and S3 each occupied by a single water. In case 2, two K^+ ions occupied the S1 and S3 sites, with the other two sites occupied by water.

MD simulations were performed using the NAMD program (Version 2.13)[14]. CHARMM36m force field was used[15]. Before sampling, each system was minimized with harmonic restraints on protein to remove bad contacts. Then the system was carefully equilibrated under harmonic restraints applied to the protein backbone at constant temperature and pressure (NPT) ensemble. The force constant started at 50 kcal/mol, and gradually decreased to 0 in 20 ns. The system was then accumulated ~400 ns, with the last 100 ns trajectory used for analysis.

To mimic the effect of action potentials on channel conformation, an external e-field of +200 mV voltage was applied to $hK_V1.3$ and $hK_V1.3$ H451N, respectively. MD simulations were performed by using the last frame from the simulation results in the absence of the applied e-field. Each trajectory was accumulated for another 100 ns.

Periodic boundary conditions were applied, with the particle mesh Ewald method used for treating long-range electrostatic interactions. Constant temperature was achieved by running Langevin dynamics, and constant pressure and surface tension were achieved by the Nose-Hoover Langevin piston method. The SHAKE algorithm was applied to constrain all bonds involving hydrogen atoms, allowing for an integration time step of 2 fs.

Configuration searching

Exhaustive searching of possible side chain configurations of H451 or N451 versus D449 were carried out, so that favorable hydrogen bonds could be formed between these two sites. In each case, the backbone atoms were fixed, and only side chains of H/N451 and D449 were allowed to rotate. To be specific, the Cα-Cβ and Cβ-Cγ bonds of these two residues were rotated every 10° one by one, and a total of ~1.68 million structures were obtained.

To assess the formation of hydrogen bonds between H451 or N451 and D449, we used two different criteria, combining of the hydrogen bond distance, $r_{donor\text{-}acceptor}$, and angle, $\theta_{donor\text{-}hydrogen\text{-}acceptor}$. Set 1 was $r \leq 3.2$ Å and $150^{\circ} \leq \theta \leq 180^{\circ}$, and set 2 was $r \leq 3.5$ Å and $150^{\circ} \leq \theta \leq 180^{\circ}$. Configurations that met these conditions were recorded except for those clashed structures in which the minimum distance between two non-bonded atoms was less than 1.2 Å.

References

1 Mastronarde, D. N. SerialEM: A Program for Automated Tilt Series Acquisition on Tecnai Microscopes Using Prediction of Specimen Position. *Microsc Microanal* **9**, 1182-1183 (2003).

2 Zheng, S. Q. *et al.* MotionCor2: anisotropic correction of beam-induced motion for improved cryo-electron microscopy. *Nat Methods* **14**, 331-332 (2017).

3 Zhang, K. Gctf: Real-time CTF determination and correction. *J Struct Biol* **193**, 1-12 (2016).

4 Zivanov, J. *et al.* New tools for automated high-resolution cryo-EM structure determination in RELION-3. *eLife* **7**, e42166 (2018).

5 Kucukelbir, A., Sigworth, F. J. & Tagare, H. D. Quantifying the local resolution of cryo-EM density maps. *Nat Methods* **11**, 63-65 (2014).

6 Emsley, P., Lohkamp, B., Scott, W. G. & Cowtan, K. Features and development of Coot. *Acta Crystallogr D Biol Crystallogr* **66**, 486-501 (2010).

7 Long, S. B., Tao, X., Campbell, E. B. & MacKinnon, R. Atomic structure of a voltage-dependent K^+ channel in a lipid membrane-like environment. *Nature* **450**, 376-382 (2007).

8 Pettersen, E. F. *et al.* UCSF Chimera--a visualization system for exploratory research and analysis. *J Comput Chem* **25**, 1605-1612 (2004).

9 Adams, P. D. *et al.* PHENIX: a comprehensive Python-based system for macromolecular structure solution. *Acta crystallographica. Section D, Biological crystallography* **66**, 213-221 (2010).

10 Afonine, P. V. *et al.* New tools for the analysis and validation of cryo-EM maps and atomic models. *Acta Crystallogr D Biol Crystallogr* **74**, 814-840 (2018).

11 Hoshi, T. & Armstrong, C. M. C-type inactivation of voltage-gated K^+ channels: pore constriction or dilation? *J Gen Physiol* **141**, 151-160 (2013).

12 Case, N. https://ncase.me/loopy/

13 Dong, H., Sharma, M., Zhou, H. X. & Cross, T. A. Glycines: role in alpha-helical membrane protein structures and a potential indicator of native conformation. *Biochemistry* **51**, 4779-4789 (2012).

14 Phillips, J. C. *et al.* Scalable molecular dynamics with NAMD. *J Comput Chem* **26**, 1781-1802 (2005).

15 Best, R. B. *et al.* Optimization of the additive CHARMM all-atom protein force field targeting improved sampling of the backbone phi, psi and side-chain chi(1) and chi(2) dihedral angles. *J Chem Theory Comput* **8**, 3257-3273 (2012).

Supplementary Figures

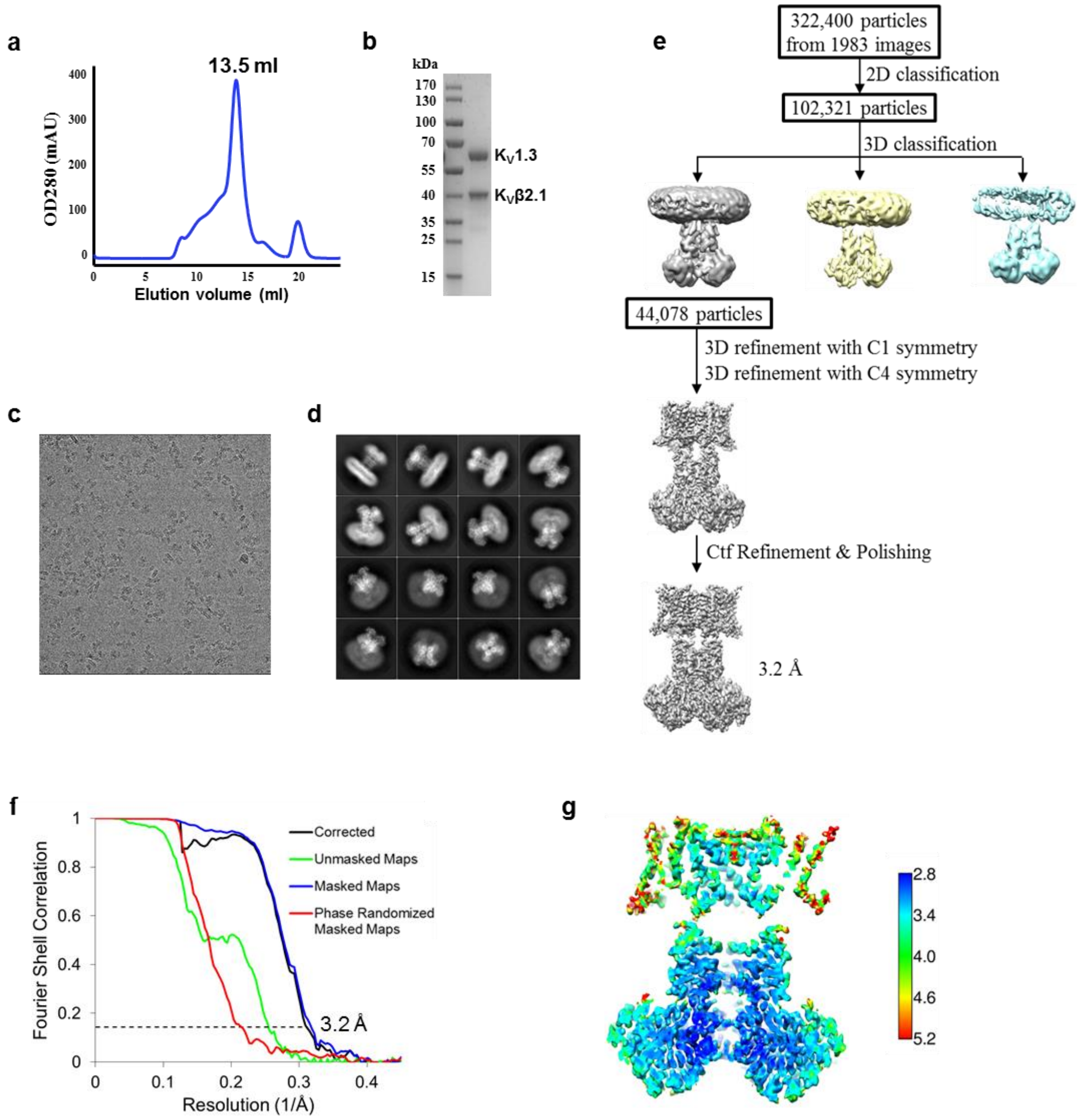

Supplementary Fig. S1. Cryo-EM structure determination of the human K_V1.3-K_Vβ2.1 complex.

a Size exclusion chromatogram of the complex. **b** SDS-PAGE of main fractions collected from size exclusion chromatography. **c, d** A representative cryo-EM micrograph (c) and representative 2D class averages (d) of the complex. **e** Cryo-EM data processing flow chart of the complex. **f** Solvent-corrected Fourier shell correlation curve from Relion 3.0 indicated that the resolution of the map for the complex was 3.2 Å at FSC=0.143. **g** EM density of the complex colored according to local resolution estimate.

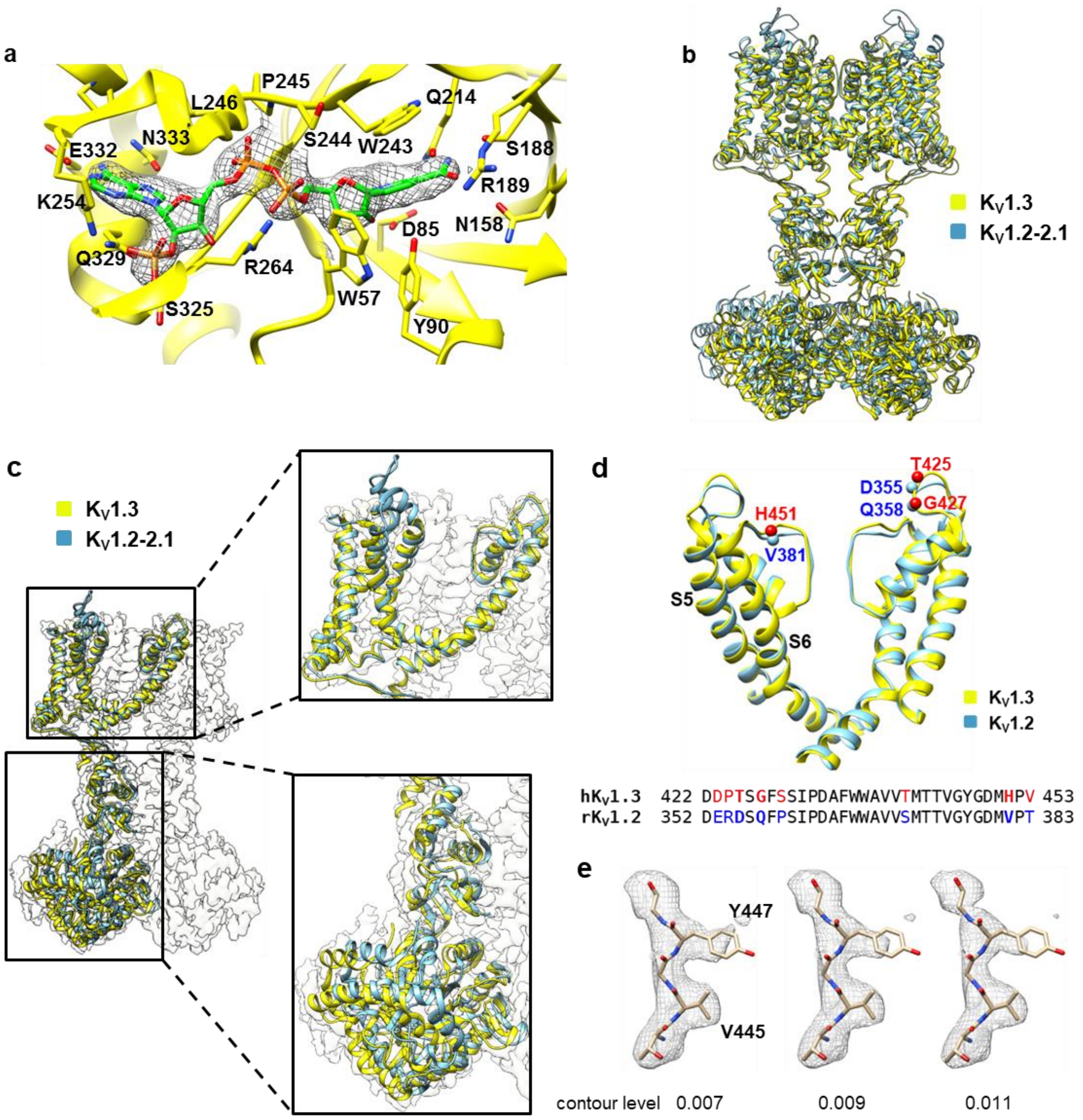

Supplementary Fig. S2. Cryo-EM structure of the human $K_V1.3$-$K_V\beta2.1$ complex.

a $NADP^+$ (green) is shown within its binding pocket in $K_V\beta2.1$. **b** Comparison of human $K_V1.3$-$K_V\beta2.1$ (yellow) and rat $K_V1.2$-2.1 chimera-$K_V\beta2$ (blue, PDB ID 2R9R) structures viewed from the membrane plane. Structures are superimposed by aligning main-chain atoms of the S5-S6 pore helices and selectivity filter. **c** Comparison of the monomers of human $K_V1.3$-$K_V\beta2.1$ (yellow) and rat $K_V1.2$-2.1 chimera-$K_V\beta2$ (blue, PDB ID 2R9R) structures with the close-up views of the transmembrane and cytosolic domains. The $K_V1.2$-2.1 chimera-$K_V\beta2$ structure is shown with fitting of the transmembrane domains into the cryo-EM map of $K_V1.3$-$K_V\beta2.1$. **d** Structural comparison of the pore domain (S5 helix, pore region and S6 helix) in human $K_V1.3$ (yellow) and rat $K_V1.2$ (blue, PDB ID 2A79). Both subunits are viewed from the membrane plane. Sequence alignment of the pore region of these two channels is shown below with residues that differ in $K_V1.3$ and $K_V1.2$ colored in red and blue, respectively. The positions of key residues in $K_V1.3$ (T425, G427 and H451) and $K_V1.2$ (D355, Q358 and V381) are shown in the structures. **e** EM densities of selectivity filter (TVGYG) in the $K_V1.3$. Weak side-chain density for Y447 is shown at different contour levels.

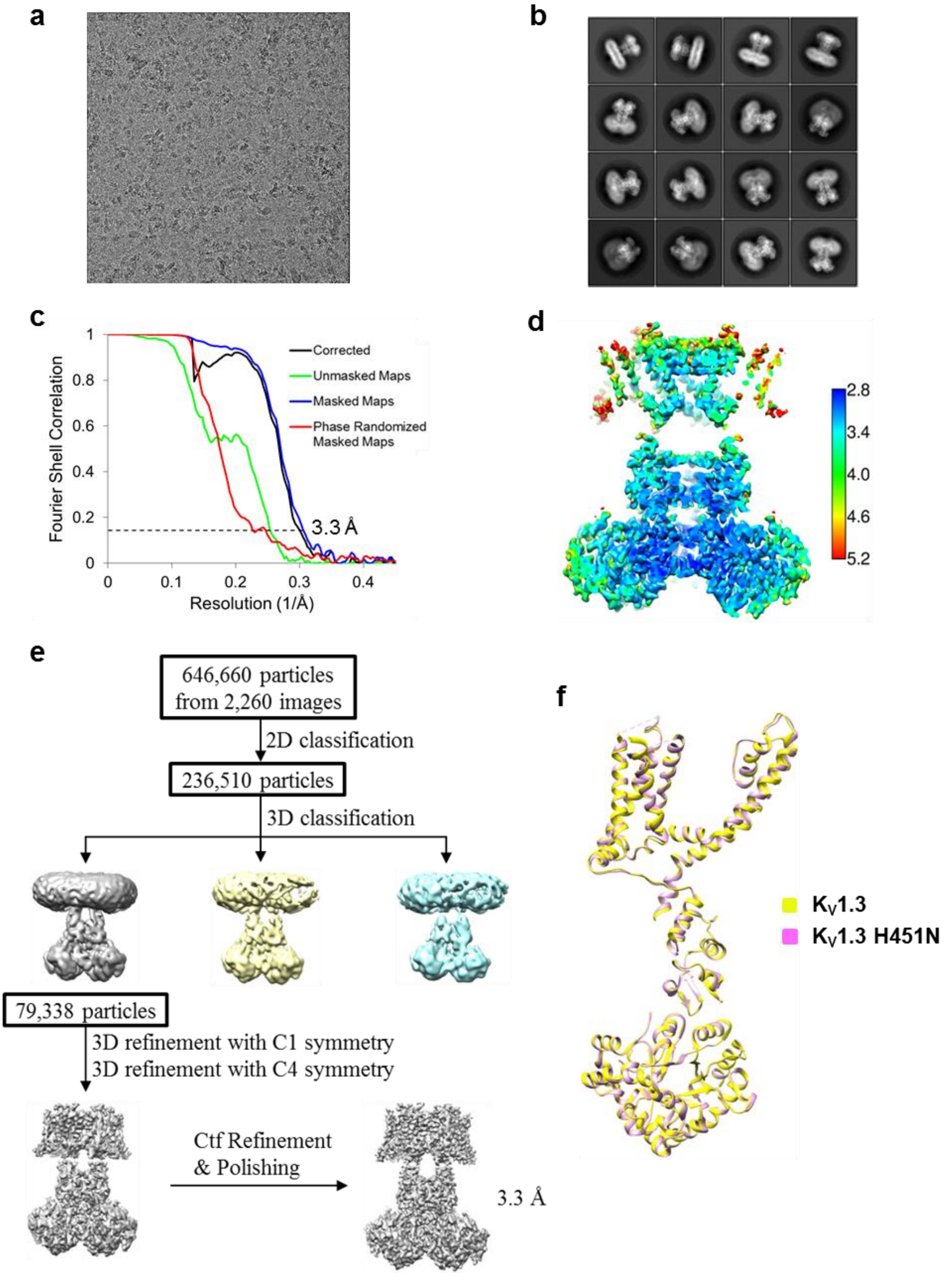

Supplementary Fig. S3. Cryo-EM structure determination of the human K_V1.3 H451N-K_Vβ2.1 complex.

a, b A representative cryo-EM micrograph (a) and representative 2D class averages (b) of the complex. **c** Solvent-corrected Fourier shell correlation curve from Relion 3.0 indicated that the resolution of the map for the complex was 3.3 Å at FSC=0.143. **d** EM map of the complex colored according to local resolution estimate. **e** Cryo-EM data processing flow chart of the complex. **f** Comparison of hK_V1.3-K_Vβ2.1 (yellow) and hK_V1.3 H451N-K_Vβ2.1 (pink) structures with monomers viewed from the membrane plane.

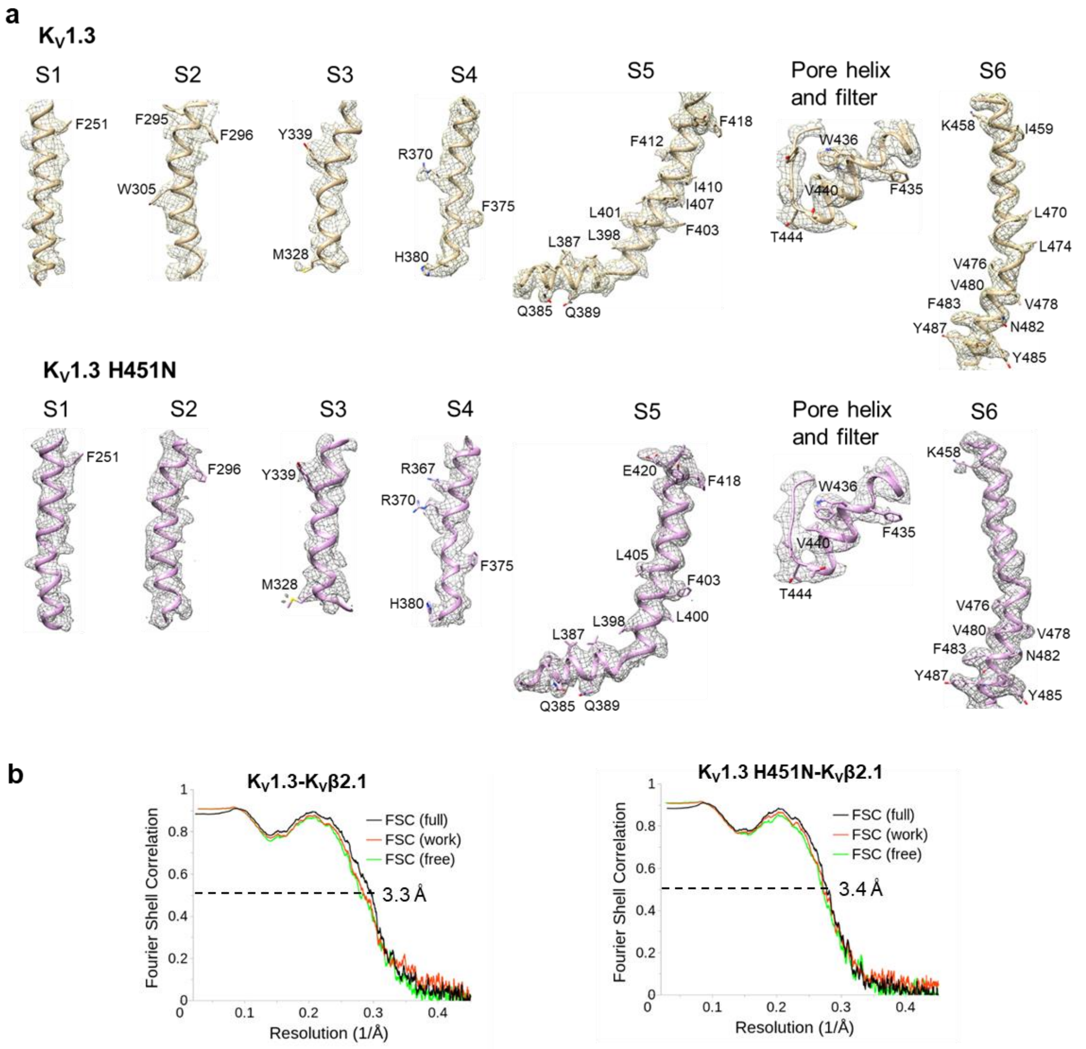

Supplementary Fig. S4. Cryo-EM maps and models for hK$_V$1.3 and the H451N mutant.

a Cryo-EM density and fitted models of the transmembrane segments of hK$_V$1.3 (top) and the H451N mutant (bottom). **b** FSC curves of the model refined against half map 1 versus the same map (red lines), of the model refined against half map 1 versus the half map 2 (green lines), and of the refined model versus the full map that the model was refined against (black lines) for hK$_V$1.3-K$_V$β2.1 (left) and hK$_V$1.3 H451N-K$_V$β2.1 (right). Dashed lines indicate 0.5 FSC value. Resolution values for the full map at 0.5 FSC are indicated beside the lines.

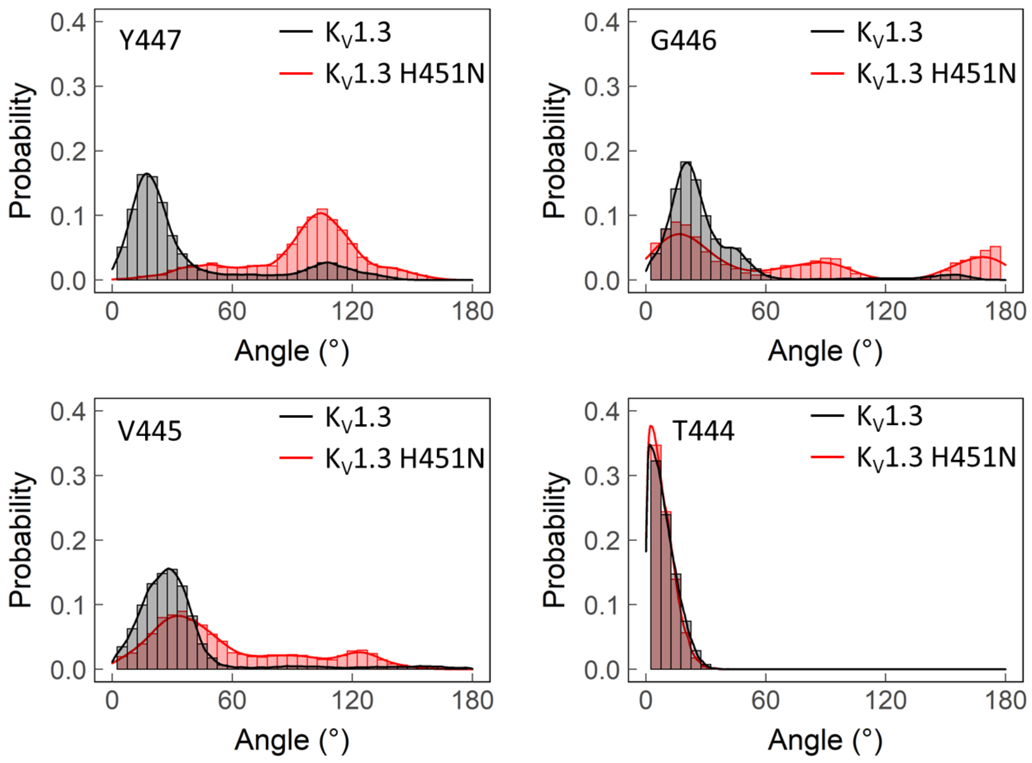

Supplementary Fig.S5. MD simulations results of the scaffold of the selectivity filter in human K_V1.3 and the H451N mutant.

The probability density of the carbonyl group's orientation on each chain was defined as the angle formed by the center of the pore, the carbonyl carbon atom and the carbonyl oxygen atom. Pore expansion, together with the carbonyl groups rotation away from the lumen at the C-terminal side, leads the deformation of the selectivity filter.

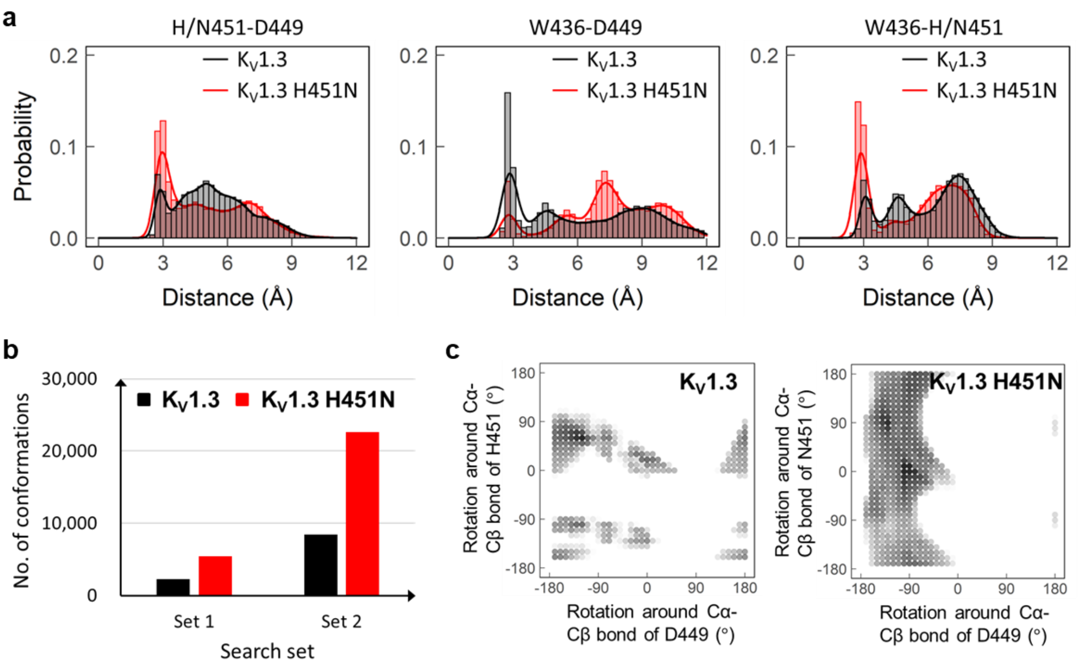

Supplementary Fig. S6. MD simulations results of the probability density of forming hydrogen bonds among H451/N451, D449 and W436 in human K$_V$1.3 and the H451N mutant.

a The normalized probability densities of hydrogen bond interactions were obtained from MD simulations on K$_V$1.3 and K$_V$1.3 H451N. The x-axis shows the distance between heavy atoms (oxygen and nitrogen) in the hydrogen bond pair. The y-axis shows the normalized probability density of forming the hydrogen bond pair labeled above the chart. **b** The population of possible H/N451-D449 interactions was obtained from exhaustive searching of the side chain configuration in both residues. Two different criteria (a combination of the hydrogen bond distance, $r_{donor\text{-}acceptor}$, and angle, $\theta_{donor\text{-}hydrogen\text{-}acceptor}$) show consistent results that the formation of N451-D449 hydrogen bond is more favorable than H451-D449. Set 1 is $r \leq 3.2$ Å, and $150^{\circ} \leq \theta \leq 180^{\circ}$, and set 2 is $r \leq 3.5$ Å, and $150^{\circ} \leq \theta \leq 180^{\circ}$. **c** The distributions of dihedral angles of residues 449 and 451 in K$_V$1.3 (left) and K$_V$1.3 H451N (right) that can form the hydrogen bond are shown. The normalized probability is in grayscale, and the darker regions have higher probability.

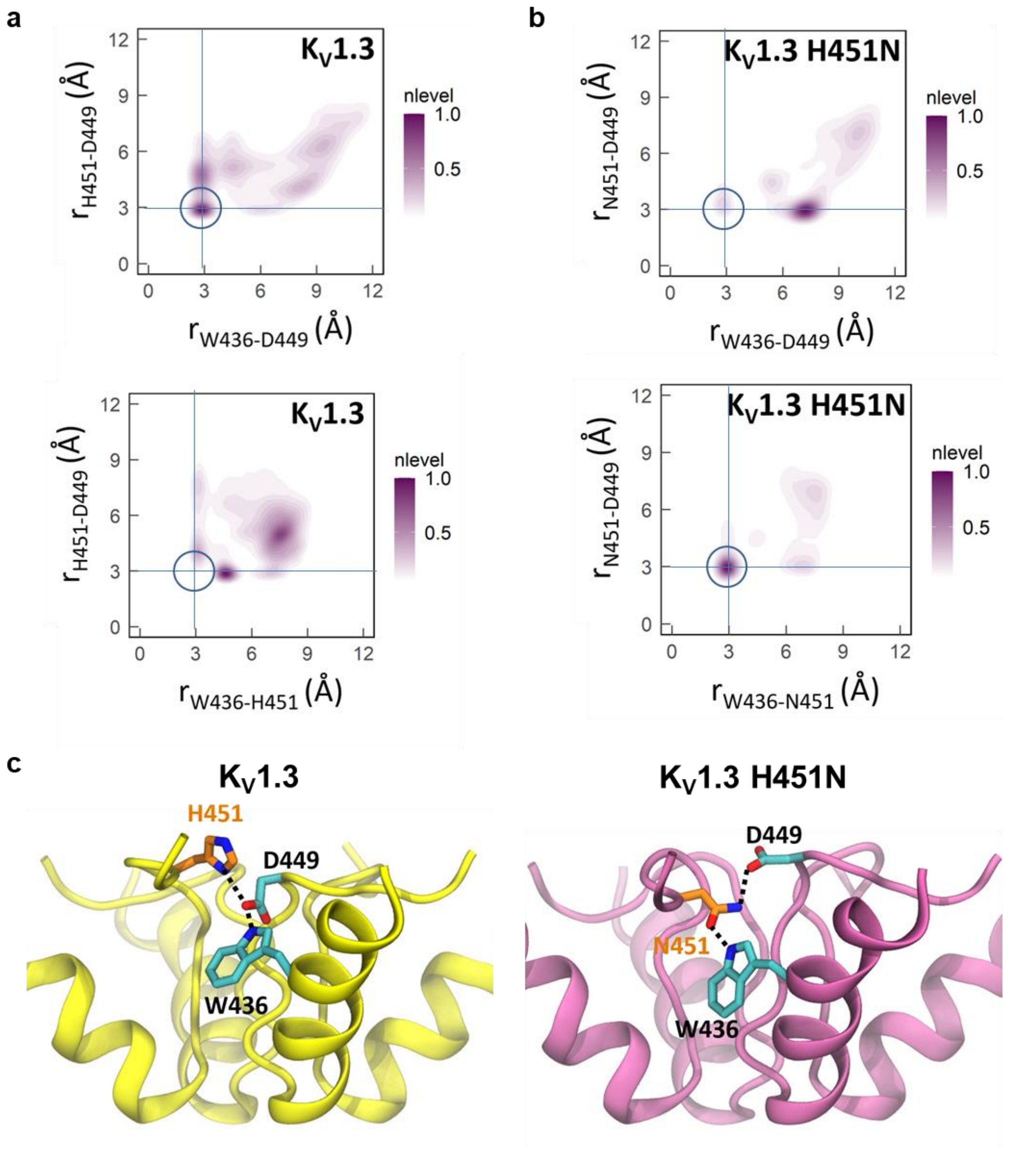

Supplementary Fig. S7. The residue at 451 site plays a key role in the C-type inactivation of human K$_V$1.3 by regulating the hydrogen bond network with W436 and D449 involved.
a, b The probability density of hydrogen bond formation is obtained from MD simulations. A dominant intra-subunit W436-D449 interaction and a secondary inter-subunit H451-D449 interaction are present in K$_V$1.3 (a). Inter-subunit N451-D449 and W436-N451 interactions are dominant in K$_V$1.3 H451N (b). Both the x-axis and y-axis show the distance between heavy atoms (oxygen and nitrogen) in the hydrogen bond pair. The lines represent the distance value of 3 Å. **c** Representative snapshots of the hydrogen bond network in K$_V$1.3 (left) and K$_V$1.3 H451N (right) are obtained from MD simulations. W436 and D449 are in the same subunit of the channel, while H451 or N451 come from the adjacent subunit. Hydrogen bonds are shown as dashed lines.

Supplementary Table S1. Statistics of cryo-EM data collection, 3D reconstruction and model refinement.

	$hK_V1.3$-$K_V\beta2.1$ (EMDB-31148) (PDB 7EJ1)	$hK_V1.3$ H451N-$K_V\beta2.1$ (EMDB-31149) (PDB 7EJ2)
**Data collection and processing**		
Magnification	29,000	29,000
Voltage (kV)	300	300
Electron exposure ($e^-/Å^2$)	62	62
Defocus range (μm)	-1.5~-2.5	-1.5~-2.5
Pixel size (Å)	1.014	1.014
Symmetry imposed	C4	C4
Initial particle images (no.)	322,400	646,660
Final particle images (no.)	44,078	79,338
Map resolution (Å)	3.2	3.3
FSC threshold	0.143	0.143
**Refinement**		
Model resolution (Å)	3.3	3.4
FSC threshold	0.5	0.5
Map sharpening *B* factor ($Å^2$)	-62	-82
Model composition		
Non-hydrogen atoms	21,236	20,968
Protein residues	2740	2728
Ligands	4 (NAP)	4 (NAP)
B factors ($Å^2$)		
Protein	89.97	101.89
Ligand	58.81 (NAP)	60.13 (NAP)
R.m.s. deviations		
Bond lengths (Å)	0.009	0.007
Bond angles (°)	0.924	0.816
Validation		
MolProbity score	1.70	1.75
Clashscore	6.51	7.49
Poor rotamers (%)	0.00	0.00
Ramachandran plot		
Favored (%)	95.01	95.07
Allowed (%)	4.84	4.78
Disallowed (%)	0.15	0.15
